# Integrated Care – Defining for the Future through the Eye of the Beholder

**DOI:** 10.5334/ijic.6427

**Published:** 2021-09-20

**Authors:** Niamh Lennox-Chhugani

**Affiliations:** 1 International Foundation for Integrated Care (IFIC), GB

**Keywords:** Definition, Integrated care, Coordination, Continuity, Population health, Prevention

The International Foundation for Integrated Care (IFIC) is about to launch its five year strategy. While coordinating this work, I have been fortunate to have conversations with many of the leading thinkers in the field of integrated care who have been part of IFIC’s journey to date. A question which has arisen numerous times is “what is IFIC’s definition of integrated care?”

It is always worth reflecting on the definition of a concept like integrated care periodically. This allows us to think about how definitions evolve in response to everyday use, changing context and evolving knowledge. Sometimes it prompts us to think about how a definition might need to change going forward.

## The history of defining integrated care

Integrated care has historically been defined conceptually by the problems it has aimed to solve: fragmentation of services, health systems not designed for the needs of an aging population living with multiple chronic diseases, and under-resourced primary care. Goodwin, Stein and Amelung [1] borrowed from Kodner [2] to describe integrated care as a polymorphous concept or one that has many different forms. More recently Hughes, Shaw and Greenhalgh [3] have described integrated care as an emergent set of practices. IFIC’s founding CEO, Nick Goodwin [4], highlighted the importance of a commitment to improving the quality and safety of care services through ongoing and co-productive partnerships lying at the heart of integrated care. A report by the European Union’s Expert Group on Health Systems Performance Assessment in 2017, which included many of IFIC’s global network, struggled to agree a common definition for integrated care. The definition they arrived at was *Integrated care includes initiatives seeking to improve outcomes of care by overcoming issues of fragmentation through linkage or co-ordination of services of providers along the continuum of care* [5].

If we go back to that seminal paper by Kodner in 2009 [2], he pointed out that the term integrated care meant different things to different people in different contexts. We have seen the term adopted widely by politicians, policymakers and practitioners globally over the last decade alongside such terms as “person-centred care”; “population health management”; “case management” and “chronic care/long term condition management” to name but a few. At IFIC, we have developed the nine pillars of integrated care, based on our many years of work and collaboration in the development of multiple frameworks. We know from many implementation studies that the interdependencies between different moving parts of any integrated care system are multiple and complex. We also know that when integration happens at the level of the person and community this is where it has greatest impact.

While there are benefits to maintaining some ambiguity around the definition of integrated care in the evolving fields of research and practice, being more precise in our definition as the International Foundation for Integrated Care will help to better guide our studies of real-world implementation, to curate the evidence effectively, and ultimately be more effective advocates of integrated care.

## Defining the features of integrated care

The central defining features of integrated care which cuts across all stakeholders (people and communities, individual providers, a system of organisations, and policymakers) are continuity and coordination. Continuity occurs temporally and coordination occurs spatially. For the person at the centre of care, their experience is seamless across formal/informal care, professional, organisational and sectoral boundaries and continuous over time. For providers, they design care to effectively manage transitions from one profession, organisation or sector to another over multiple episodes of care. For policymakers, integrated care requires them to ensure that the wider context supports continuity and coordination and does not work against them.

Continuity and coordination alone require multiple interventions to be put in place. Our health and care systems have come to rely on specialisation and compartmentalisation [6]. This approach to design has introduced fragmentation across time and space and all the risks that come with that. Changing this century-old approach to designing health and care systems is no small feat. If we focus just on developing coordination and continuity alone, we need to change how we govern and organise services, resource them with a new set of competencies, regulate them and fund them.

## Expanding the defining features of integrated care

The focus more recently on the triple aim of health and care systems and the use of terminology like population health management, has prompted some proponents of integrated care to broaden the definition of integrated care to include population health management and extend the reach of integrated care to public health interventions including prevention. This is where context becomes more influential. Health and care system history and maturity will determine whether this is desirable or culturally acceptable.

An example of the recognition of dependencies between integrated care and public health can be seen in the initiatives put in place in England over the last 6 years (Lennox-Chhugani, 2021) [7] to develop integrated care systems. Integrated care interventions are part of a population health management approach alongside public health interventions. Integrated care interventions focus on specific populations experiencing a need for care. Sometimes we delineate those populations by primary long term or chronic condition (e.g. dementia), sometimes by an over-arching mosaic of chronic conditions (e.g. frailty). Public health interventions manage and reduce health risks across an entire geographic, risk assessed or age-based population (e.g. diabetes prevention). We need both of these to act in concert to deliver the best health and well-being outcomes for populations.

## Refining for less mature health and care systems

So far, the focus for integrated care has been on solving the problems inherent in relatively well-resourced health and care systems most in evidence in North America, Western Europe, Australia and New Zealand. But what of other regions and countries? The World Health Organisations’ Framework on integrated people-centred health services (IPCHS) in 2016 [8] was intended to be global but in practice the shift towards integrated care remains concentrated in more mature and better funded systems.

Nonetheless the central defining concepts of coordination and continuity resonate in other contexts. Scarcity of resources, limited access to services, and issues of trust between the population and health and care providers are additional challenges but equally present opportunities to redesign health and care systems that already rely heavily on carers and communities for the provision of services. We are starting to see evidence of this work in Latin America and The Caribbean.

## Conclusion

Integrated care includes the word ‘care’ and one can argue that this implies a person has entered a ‘care’ system by virtue of having a specific health or care need. Not all members of a community or population will be in such a system at a given point in time. Population health management allows health and care systems shape and influence the social determinants of population health and seeks to prevent people entering into this system of ‘care’ in the first place or minimising their need for care while living with a long-term condition.

Redesigning our health and care systems involves considerable disruption for health and care professionals and the people and the communities in which they live. Focusing on the central defining features of integrated care, helps us to understand its benefits, and how these compare to the resource and opportunity costs associated with redesign.

We can then explore what impact integrated care initiatives have in the context of a wider population health approach. Implementing person-centred integrated care that builds on community assets and strengths in less mature health and care systems will inform our practice in more mature systems. It will also ensure we deliver the promise of integrated care that improves global health and wellbeing.

The work of defining integrated care will never be finished. Everything about the landscapes in which we are living are changing constantly. With this change, our definition of integrated care will evolve. One day, we at IFIC hope that it will be *lingua franca* of health and care systems everywhere. As a linguist at the risk of over-simplifying Wittgenstein, ‘in most cases, the meaning of a word is its use’ [9].
